# Delayed sternal closure for intractable bleeding after acute type A aortic dissection repair: outcomes and risk factors analyses

**DOI:** 10.1186/s13019-022-01946-z

**Published:** 2022-08-19

**Authors:** Chun-Yu Lin, Meng-Yu Wu, Chi-Nan Tseng, Hsin-Fu Lee, Feng-Chun Tsai

**Affiliations:** 1 Department of Medicine, College of Medicine, Chang Gung University, Taoyuan City, Taiwan; 2 Department of Cardiothoracic and Vascular Surgery, New Taipei Municipal TuCheng Hospital, No.6, Sec.2, JinCheng Rd, TuCheng, New Taipei City, 236 Taiwan; 3 Department of Cardiothoracic and Vascular Surgery, Linkou Medical Center, Chang Gung Memorial Hospital, Taoyuan City, Taiwan; 4 Department of Cardiology, New Taipei Municipal TuCheng Hospital, New Taipei City, Taiwan

**Keywords:** Acute type A aortic dissection, Bleeding, Coagulopathy, Delayed sternal closure, Mediastinal packing

## Abstract

**Background:**

Perioperative coagulopathy and intractable bleeding are severe complications in acute type A aortic dissection (ATAAD) repair surgery. Mediastinal packing with delayed sternal closure (DSC) is a commonly adapted technique to stabilize the hemorrhagic tendency. This retrospective study aims to investigate the early and late outcomes and risk factors in patients who underwent DSC procedure during ATAAD repair surgery.

**Methods:**

This study investigated 704 consecutive patients who underwent ATAAD repair at this institution between January 2007 and September 2020. These patients were dichotomized into the DSC (n = 109; 15.5%) and primary sternal closure (PSC) groups (n = 595; 84.5%). The clinical features, surgical information, postoperative complications, 5-years cumulative survival, and freedom from reoperation rates were compared. A multivariate logistic regression analysis was used to identify the independent risk factors for patients who underwent DSC.

**Results:**

The DSC group showed a higher rate of hemopericardium and preoperative malperfusion, and was associated with longer cardiopulmonary bypass and aortic clamping times and a higher rate of intraoperative extracorporeal membrane oxygenation (ECMO) support. The DSC group showed higher blood transfusion volumes and rate of reexploration for bleeding after surgery. However, the in-hospital mortality rates (17.4% vs. 13.3%; *P* = 0.249), 5-year survival rates (66.9% vs. 68.2%; *P* = 0.635), and freedom from reoperation rates (89.1% vs. 82.5%; *P* = 0.344) were comparable between the DSC and PSC groups. Multivariate analysis revealed that hemopericardium, preoperative malperfusion, and intraoperative ECMO support were risk factors for implementing DSC.

**Conclusions:**

DSC is an efficient life-saving technique to stabilize patients with intractable bleeding after undergoing ATAAD repair surgery, which leads to acceptable short- and long-term outcomes. Patients who were at risk for intractable bleeding should have early decision-making for implementing DSC.

## Introduction

Stanford acute type A aortic dissection (ATAAD) requires prompt surgical treatment for life-saving and is associated with high morbidity and mortality rates [1, 2]. Postoperative bleeding and surgical reoperation for bleeding tendency are one of the feared complications [3–5]. The consumption coagulopathy was commonly observed in patients undergoing ATAAD repair surgery, which was induced by the persistent systemic activation of the coagulation system and consumption of clotting factors from intravascular thrombosis of the dissected false lumen [5]. In addition, prolonged implementation of cardiopulmonary bypass (CPB), extensive tissue trauma due to complex aortic repair procedures, and massive blood transfusion would exacerbate the coagulation deficiencies further. Mediastinal packing with delayed sternal closure (DSC) was a commonly adapted technique for managing intractable perioperative bleeding, which efficiently stabilized the hemorrhagic tendency following complex cardiac and proximal aortic surgeries [6–8]. However, a detailed investigation of the outcomes and associated risk factors for DSC among the ATAAD population was not reported in previous literatures. In this study, we conducted a retrospective analysis with the database from an individual aortic surgery center to compare the individual characteristics, and early and late outcomes between patients who had DSC and primary sternal closure (PSC) after ATAAD repair surgery.

## Materials and Methods

### Patient enrollment and preoperative management

**Fig. 1:**
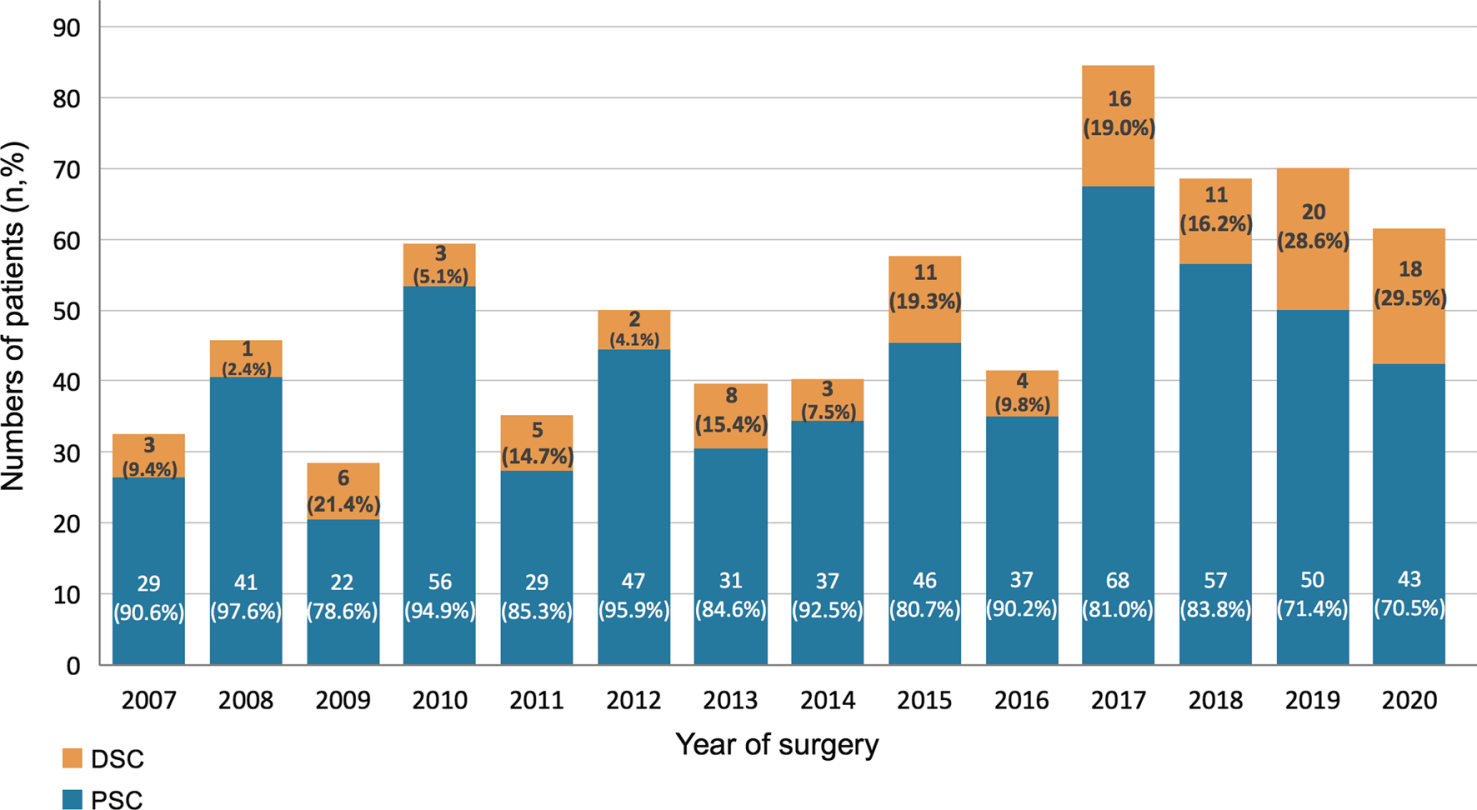
Annual distribution for patients of overall ATAAD, DSC, and PSC during the study period. ATAAD, acute type A aortic dissection; DSC, delayed sternal closure; PSC, primary sternal closure

The study protocol was approved by the Institutional Review Board of Chang Gung Medical Foundation (approval number 202200020B0). All data were accessed using the institutional database of aortic dissection. This study investigated a total of 704 consecutive adult patients who underwent emergency ATAAD repair between January 2007 and September 2020 at this institution. All patients were diagnosed via helical computed tomography at the emergency department. Following the confirmation of an ATAAD diagnosis, patients were immediately transferred to the operating room for undergoing further aortic repair surgeries. The 704 included patients were dichotomized into the DSC (n = 109; 15.5%) and PSC (n = 595; 84.5%) groups based on the implementation of mediastinal packing for intractable intraoperative bleeding. The annual cases of the overall cohort, DSC, and PSC groups during the study period are illustrated in Fig. 1. The patients’ preoperative hemodynamics were stabilized with intravenous beta-blockers to maintain a systolic blood pressure < 120 mmHg and heart rate of 60–70 bpm according to the established guidelines [9]. For patients who presented with shock status, medical and surgical resuscitation procedures were applied based on the standardized protocols in this institute [10, 11].

### Aortic dissection repair procedures

The technical principles of ATAAD repair procedures were discussed in previous studies [12, 13]. Double artery cannulation using a combination of right axillary and femoral arterial access and the antegrade cerebral perfusion (ACP) strategy were commonly used for patients with a relatively stable preoperative condition. In contrast, isolated femoral artery cannulation with retrograde cerebral perfusion (RCP) was preferred for patients who presented with unstable condition. Following sternotomy, the right atrium or vena cava was cannulated, and CPB with systemic hypothermia was initiated. In general, the dissected aorta was replaced with Dacron prosthetic grafts based on the site of the primary proximal entry tear and clinical presentation. The tubular ascending aorta was routinely resected and the proximal anastomosis was usually performed first, followed by open distal anastomosis under deep hypothermic circulatory arrest (18–22 °C). All graft-aorta anastomoses were reinforced with Teflon strips. During circulatory arrest, the femoral arterial flow was temporarily suspended and selective ACP through the right axillary artery or RCP through the superior vena cava was performed depending on the selected cannulation strategy. Concomitant aortic root replacement with a composite Valsava graft and frozen elephant trunk procedure with a covered stent-graft were performed if the extent of aortic dissection involved the aortic root and descending thoracic aorta, respectively. All patients were transferred to a specialized cardiovascular intensive care unit (ICU) for postoperative treatment and observation after undergoing ATAAD repair.

### Mediastinal packing and delayed sternal closure procedures

All graft-aorta anastomoses, cannulation sites, and tissue rough surface were carefully examined and the active bleeders were reinforced with pledgeted compression sutures before weaning from CPB. After termination of CPB with protamine administration and maximal surgical hemostasis, patients with any of the following four criteria were defined as intractable perioperative bleeding and DSC was performed: bleeding exceeded 50 ml within 30 min; administration of more than 8 units of red blood cells; uncontrolled bleeding from the inaccessible bleeding sites; presence of hemodynamic instability if sternal closure was attempted. The saline-moistened sterile gauzes (Kerlix AMD*,* Covidien*,* St. Louis, MO, USA) were used to extensively pressurize the uncontrollable bleeding sites. The Prosthetic aortic grafts were surroundedly compressed with sterile gauzes first and followed by packing the residual mediastinal space. The pericardium and sternum were left open and the skin wound was covered with a plastic patch made by sterile intravenous fluid bag. Broad*-*spectrum intravenous antibiotics were regularly administered for preventing mediastinal and wound infections. The platelet count was maintained above 80,000–100,000 mm^3^ and fibrinogen level was maintained above 150 mg/dL. Prothrombin time and activated partial thromboplastin time levels were also closely monitored, and coagulopathy was reversed by plasma transfusion. Mediastinal reexploration was usually performed within 24–48 h at the operating room if the hemodynamics were stable and bleeding tendency improved (blood loss from chest tubes < 600 ml and transfusion of red blood cells < 4 units for at least 12 consecutive hours). The blood clot was evacuated, and all identified bleeders were carefully rechecked. Sternal closure was performed with stainless steel wires if hemostasis has been well achieved. Prophylactic broad-spectrum intravenous antibiotics were continued for at least 1–2 weeks with a close observation of signs of subsequent infection.

### Statistical analyses

Statistical analyses were performed using SPSS for Windows (version 22.0; IBM Corp., Armonk, NY, USA). Data were presented as means ± standard deviation and as numbers and percentages for numerical and categorical variables, respectively. To compare the intergroup disparities between the DSC and PSC groups, the chi-square test for categorical variables and the independent t-test for numerical variables were used, respectively. A multivariate logistic regression analysis was used to identify the independent risk factors of patients who underwent DSC. Preoperative and surgical variables listed in Tables 1 and 2 were tested by the univariate logistic regression analysis first. The variables with a *P* < 0.05 in the univariate logistic regression analysis were further analyzed via the multivariate logistic regression analysis. The Kaplan–Meier method was used to estimate the 5-year cumulative survival and freedom from aortic reoperation rates of the two groups, which were compared using the log-rank test. For all analyses, the statistical significance was set at *P* < 0.05.

**Table 1:** Preoperative characteristics

Parameters	Total	DSC	PSC	*P-*value
	n = 704	n = 109	n = 595	
*Clinical demographics*				
Age (years)	56.6 ± 13.7	57.1 ± 14.5	56.6 ± 13.5	0.716
Sex (female, n,%)	214, 30.4	34, 31.2	180, 30.3	0.844
Hypertension (n,%)	510, 72.4	75, 68.8	435, 73.1	0.355
Diabetes mellitus (n,%)	47, 6.7	5, 4.6	42, 7.1	0.342
Creatinine (mg/dL)	1.4 ± 1.4	1.6 ± 1.4	1.4 ± 1.4	0.157
End-stage renal disease (n,%)	12, 1.7	1, 0.9	11, 1.8	0.490
*Preoperative condition*				
Systolic blood pressure (mmHg)	97.6 ± 19.7	96.7 ± 21.2	97.8 ± 19.4	0.600
Systolic blood pressure < 90 mmHg (n,%)	162, 23.0	29, 26.6	133, 22.4	0.332
Cardiopulmonary resuscitation (n,%)	23, 3.3	5, 4.6	18, 3.0	0.399
Ventilator support (n,%)	39, 5.5	10, 9.2	29, 4.9	0.071
Repeat surgery (n,%)	25, 3.6	4, 3.7	21, 3.5	0.942
*Clinical presentation*				
DeBakey type II (n,%)	68, 9.7	10, 9.2	58, 9.7	0.852
Intramural hematoma (n,%)	132, 18.8	21, 19.3	111, 18.7	0.881
Chest/back pain (n,%)	525, 74.6	83, 76.1	442, 74.3	0.682
Hemopericardium (n,%)	236, 33.5	47, 43.1	189, 31.8	0.021
Aortic regurgitation > moderate (n,%)	104, 14.8	16, 14.7	88, 14.8	0.976
Malperfusion^a^ (n,%)	109, 15.5	29, 26.6	80, 13.4	< 0.001
Acute myocardial infarction (n,%)	15, 2.1	5, 4.6	10, 1.7	0.053
Cerebral infarction (n,%)	29, 4.1	9, 8.3	20, 3.4	0.018

*DSC* delayed sternal closure; *PSC* primary sternal closure

^a^Occurrence of preoperative limb ischemia, cerebral infarction, paraplegia, coronary artery occlusion, and mesenteric ischemia

**Table 2:** Surgical information

Parameters	Total	DSC	PSC	*P-*value
	n = 704	n = 109	n = 595	
Femoral artery cannulation (n,%)	667, 94.7	103, 94.5	564, 94.8	0.899
Axillary artery cannulation (n,%)	587, 83.4	90, 82.6	497, 83.5	0.804
AsAo cannulation (n,%)	1, 0.1	0	1, 0.2	0.668
*Aortic repair procedures*				
Isolated AsAo replacement (n,%)	443, 62.9	63, 57.8	380, 63.9	0.228
Root replacement (n,%)	76, 10.8	17, 15.6	59, 9.9	0.079
Arch replacement (n,%)	199, 28.3	31, 28.4	168, 28.2	0.965
Partial arch (n,%)	128, 18.2	21, 19.3	107, 18.0	0.750
Total arch (n,%)	71, 10.1	10, 9.2	61, 10.3	0.731
Frozen elephant trunk (n,%)	57, 8.1	12, 11.0	45, 7.6	0.225
Resection of primary entry tear (n,%)	503, 71.4	82, 75.2	421, 70.8	0.342
Cardiopulmonary bypass time (min)	256.0 ± 77.7	278.4 ± 86.8	251.9 ± 75.3	0.001
Aortic clamping time (min)	166.4 ± 54.3	181.8 ± 65.1	163.6 ± 51.6	0.007
Circulatory arrest time (min)	50.8 ± 24.2	52.8 ± 26.2	50.4 ± 23.8	0.357
Hypothermia temperature (°C)	20.4 ± 2.5	20.2 ± 2.4	20.4 ± 2.5	0.432
Antegrade cerebral perfusion (n,%)	597, 84.8	97, 89.0	500, 84.0	0.185
Retrograde cerebral perfusion (n,%)	107, 15.2	12, 11.0	95, 16.0	0.185
Extracorporeal membrane oxygenation (n,%)	23, 3.3	11, 10.1	12, 2.0	< 0.001

*AsAo* ascending aorta; *DSC* delayed sternal closure; *PSC* primary sternal closure

## Results

### Patient demographics

Table 1 shows the preoperative demographics, which did not present with significant differences in age, sex, comorbidities, and preoperative conditions. Overall, the mean age was 56.6 ± 13.7 years, and 30.4% of patients were females. Chest or back pain was the most common symptom of ATAAD, which accounted for more than 70% of patients in both groups. Higher rates of hemopericardium (43.1% vs. 31.8%; *P* = 0.021), end-organ malperfusion (26.6% vs. 13.4%; *P* < 0.001), and cerebral infarction (8.3% vs. 3.4%; *P* = 0.018) were found in the DSC group.

### Surgical information

Table 2 shows the detailed information on surgical variables. No significant differences on vascular access of cannulation, extents of aortic repair procedure, and rates of primary entry tear resection were found between two groups. The DSC group showed longer time spans of CPB (278.4 ± 86.8 vs. 251.9 ± 75.3 min; *P* = 0.001) and aortic cross-clamping (181.8 ± 65.1 vs. 163.6 ± 51.6 min; *P* = 0.007) as compared to the PSC group. A higher rate of intraoperative extracorporeal membrane oxygenation (ECMO) support (10.1% vs. 2.0%; *P* < 0.001) was found in the DSC group.

### Postoperative complications

Table 3 shows that the in-hospital mortality rates had no significant difference between the DSC and PSC groups (17.4% vs. 13.3%; *P* = 0.249). However, the blood transfusion volumes and rate of reexploration for bleeding were both higher in the DSC group. Furthermore, the DSC group had higher rates of postoperative complications, including renal failure with hemodialysis, mesenteric ischemia, pneumonia, and prolonged ventilator support. The length of ICU stays and overall hospital course were generally longer in the DSC group, although the statistical significances were not reached.

**Table 3:** Postoperative mortality and morbidity

Parameters	Total	DSC	PSC	*P-*value
	n = 704	n = 109	n = 595	
In-hospital mortality (n,%)	98, 13.9	19, 17.4	79, 13.3	0.249
Bleeding (n,%)	18, 2.6	5, 4.6	13, 2.2	0.144
Myocardial failure (n,%)	42, 6.0	6, 5.5	36, 6.1	0.825
Brain stem failure (n,%)	18, 2.6	4, 3.7	14, 2.4	0.423
Sepsis (n,%)	20, 2.8	4, 3.7	16, 2.7	0.571
*Transfusion within 24 h after surgery*				
RBC^a^ (units)	8.9 ± 7.7	13.9 ± 11.8	8.0 ± 6.3	< 0.001
Plasma^b^ (units)	7.9 ± 6.8	12.6 ± 9.9	7.1 ± 5.6	< 0.001
Platelet (units)	19.2 ± 13.4	29.1 ± 19.8	17.4 ± 11.0	< 0.001
Reexploration for bleeding (n,%)	102, 14.5	39, 35.8	63, 10.6	< 0.001
Delirium (n,%)	125, 17.8	19, 17.4	106. 17.8	0.923
Seizure (n,%)	54, 7.7	6, 5.5	48, 8.1	0.355
Brain stroke (n,%)	116, 16.5	23, 21.1	93, 15.6	0.157
Infarction (n,%)	95, 13.5	17, 15.6	78, 13.1	0.485
Hemorrhage (n,%)	21, 3.0	6, 5.5	15, 2.5	0.092
Renal failure (n,%)	66, 9.4	16, 14.7	50, 8.4	0.039
Mesenteric ischemia (n,%)	18, 2.6	6, 5.5	12, 2.0	0.034
Limb ischemia (n,%)	20, 2.8	3, 2.8	17, 2.9	0.952
Pneumonia (n,%)	87, 12.4	22, 20.2	65, 10.9	0.007
Deep sternal wound infection (n,%)	21, 3.0	5, 4.6	16, 2.7	0.284
Extubation time (hrs)	97.9 ± 259.5	156.2 ± 478.9	87.2 ± 193.1	0.141
Ventilator support > 72 h (n,%)	226, 32.1	53, 48.6	173, 29.1	< 0.001
ICU stay (days)	7.4 ± 13.9	10.6 ± 20.8	6.9 ± 12.1	0.072
Hospital stay (days)	25.3 ± 29.2	28.9 ± 32.3	24.7 ± 28.6	0.162

*DSC* delayed sternal closure; *ICU* intensive care unit; *PSC* primary sternal closure

^a^Red blood cell transfusion including the amount of whole blood and packed red blood cell concentrate

^b^Plasma transfusion including the amount of fresh-frozen plasma and cryoprecipitate

### Risk factors associated with delay sternal closure

Table 4 shows the results of logistic regression analyses for patients at risks of undergoing DSC. Three significant risk factors for DSC were identified: preoperative hemopericardium (odds ratio [OR], 1.65; 95% confidence interval [CI] 1.07–2.55; *P* = 0.022), preoperative malperfusion (OR, 2.03; 95% CI 1.14–3.63; *P* = 0.017), and intraoperative implementation of ECMO support (OR, 4.33; 95% CI 1.70–11.05; *P* = 0.002).

**Table 4:** Logistic regression analyses for delayed sternal closure

Parameters	β-coefficient	Standard error	Odds ratio, 95% CI	*P-*value
*Univariate logistic regression*				
Hemopericardium	0.488	0.213	1.63 (1.07–2.47)	0.022
Malperfusion	0.847	0.248	2.33 (1.44–3.79)	0.001
Cerebral infarction	0.951	0.416	2.59 (1.15–5.85)	0.022
Cardiopulmonary bypass time	0.004	0.001	1.01 (1.00–1.02)	0.002
Aortic clamping time	0.006	0.002	1.01 (1.00–1.01)	0.002
Extracorporeal membrane oxygenation	1.696	0.431	5.45 (2.34–12.70)	< 0.001
*Multivariate logistic regression*				
Hemopericardium	0.503	0.220	1.65 (1.07–2.55)	0.022
Malperfusion	0.707	0.296	2.03 (1.14–3.63)	0.017
Extracorporeal membrane oxygenation	1.467	0.478	4.33 (1.70–11.05)	0.002

*CI* confidence interval

### Cumulative 5-year survival and freedom from reoperation rates

**Fig. 2:**
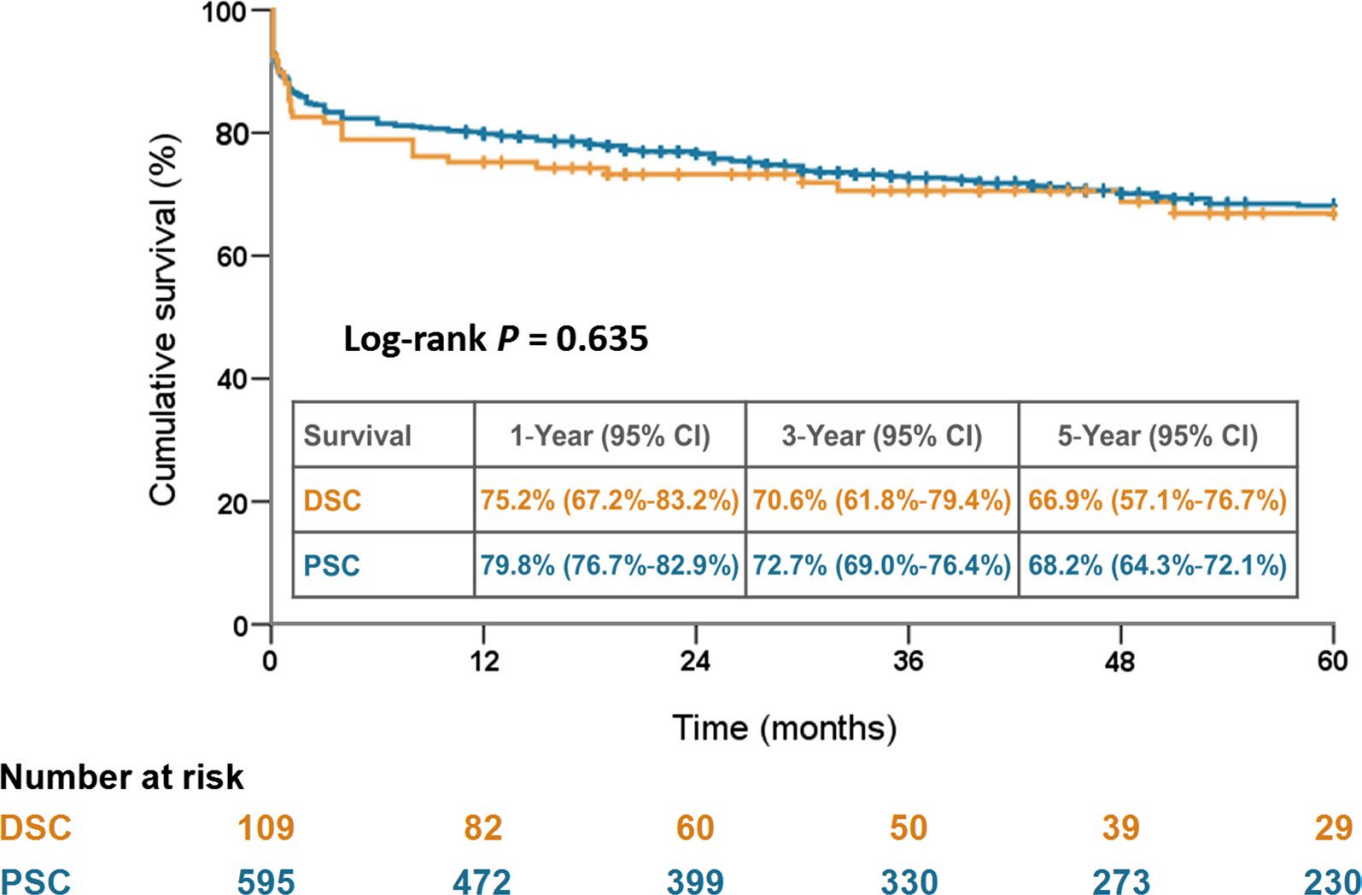
Five-year cumulative survival rates stratified by DSC and PSC. DSC, delayed sternal closure; PSC, primary sternal closure

**Fig. 3:**
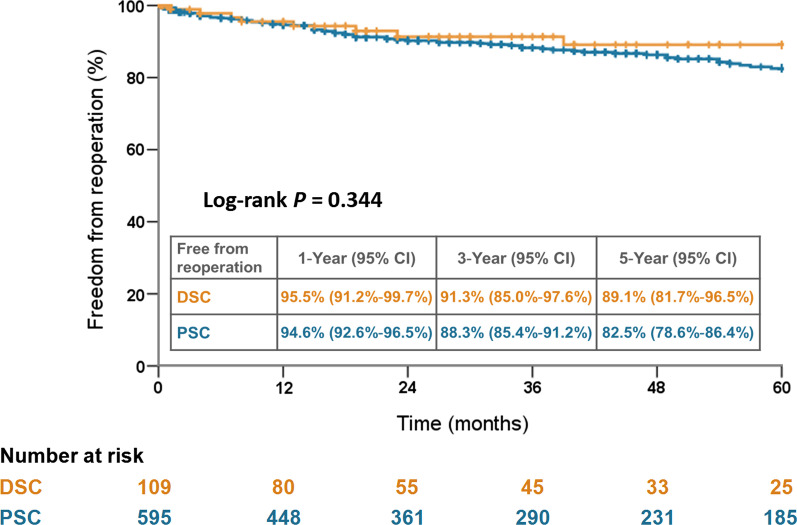
Five-year freedom from aortic reoperation rates stratified by DSC and PSC. DSC, delayed sternal closure; PSC, primary sternal closure

The averaged duration of follow-up was 4.8 ± 3.7 years (median, 4.1; range, 0.1–14.9 years). As illustrated in Fig. 2 and Fig. 3, respectively, the 5-year cumulative survival rates (66.9% vs. 68.2%; *P* = 0.635) and freedom from reoperation rates (89.1% vs. 82.5%; *P* = 0.344) were both comparable between the DSC and PSC groups.

## Discussion

The technique of DSC following open cardiac surgery was introduced in early 1980s [14], and has been served as a commonly used life-saving measure for complex cardiovascular surgery complicated with intractable bleeding [6–8]. However, the clinical outcomes and associated risk factors for DSC in the ATAAD population was scarcely reported in previous literatures. In this single-center cohort study, we investigated 704 consecutive patients (109 with DSC and 595 with PSC) who underwent ATAAD repair surgery during the study period. This study yielded several main findings. First, the incidence of DSC implementation (15.5%) was considerable in the ATAAD population owing to the effect of coagulopathy induced by the pathophysiology of disease itself and complex surgical procedures. Second, DSC were found to have acceptable short- and long-term outcomes as compared to those with PSC in carefully selected patients. Third, patients who had preoperative hemopericardium, preoperative malperfusion, and intraoperative implementation of ECMO are at risk for intractable perioperative bleeding and should be carefully evaluated for implementing DSC.

The dysfunction of coagulation system was commonly observed in patients with acute aortic dissection, especially in patients who underwent ATAAD repair surgery. The pathology and associated mechanisms were multifactorial, including the activation of the coagulation and fibrinolytic systems for intravascular thrombosis in false lumen of the dissected aortic segment, which leads to reduction in clotting factors and dysfunction of platelet. Furthermore, intraoperative administration of heparin, prolonged CPB duration, and extensive tissue trauma for the complex aortic repair procedures were also associated with the coagulopathy. Despite the improvements of management algorithms, surgical strategies, and CPB techniques in the last few decades, reoperation for postoperative bleeding remains as a common and dangerous complication, which ranges from 9 to 20% for the ATAAD population according to different international data registries [15–17]. The recovery process of aortic dissection-related coagulation defects may last more than 24 h from the onset of acute aortic dissection [3]. Therefore, the application of mediastinal packing with DSC would be life-saving to minimize the bleeding tendency and temporarily maintain the hemodynamic stability.

Although the in-hospital mortality rates were similar between the DSC and PSC groups in this study, the DSC group showed higher postoperative complication rates, including massive blood transfusion, reexploration for bleeding, acute renal failure, and prolonged ventilator support and ICU stay. There are several interpretations to these findings. First, even with a pressurized packing for inaccessible bleeding sites, some minor bleeding from the needle holes and fragile tissue could still present. In addition, the residual pericardial space has decreased owing to the extensive impaction of gauzes, which may reduce hemodynamic tolerability to cardiac tamponade. We suggest that a more aggressive strategy of reexploration for bleeding should be applied in patients who presented with persistent bleeding or signs of cardiac tamponade after undergoing mediastinal packing with DSC procedures. Second, managements such as restoration of body temperature, reversing metabolic imbalance, and aggressive blood transfusion would be mandatory after surgery for correction of coagulation deficiency. However, the massive blood transfusion may be associated with considerable injuries to the organs. Furthermore, the DSC group showed higher transfusion volumes for all kinds of blood products in this study. This might also represent a more severe blood loss and inadequate systemic perfusion status, which could lead to organ malperfusion. In previous studies reported by Wu and Helgason et al., massive blood transfusion was defined as one of the main risk factors for postoperative acute renal injury and renal replacement therapy [18, 19]. The present study demonstrated a similar finding. In this institute, if acute renal failure develops after ATAAD surgery, the renal replacement therapy was promptly implemented to reduce the negative impact of fluid overload, metabolic acidosis, and electrolyte imbalance based on the Acute Kidney Injury Network criteria [20]. Lastly, considering the higher complication rates mentioned above in the DSC group, prolonged time spans of ventilator support and ICU stay may also be expected.

The associated mediastinal and sternal wound infections remain one of the major concerns following DSC. However, Fanning and Boeken et al. reported that DSC did not yield an increased incidence of mediastinal infection as compared to PSC procedure [21, 22]. Similar outcomes were observed in the present study; patients who underwent DSC and PSC had comparable deep sternal wound infection rates. In addition to the standardized procedures of DSC and routine prophylactic antibiotics to effectively reduce the risk of infection, we suggest that patients with DSC should also have intensive surveillance for early detection and treatment of mediastinal infection.

### Limitations

This study has several limitations. First, as a retrospective and non-randomized control study, potential bias may have existed that could influence the homogeneity of the DSC and PSC groups. Second, the principles of implementing the DSC procedure were based on the institutional consensus. However, the final decision making was left to the discretion of the operating surgeon with full consideration of each individual patient’s clinical condition. Therefore, DSC procedure may be implemented more aggressively in some patients with potential bleeding risks, such as those with prior cardiac surgery, fragile aortic tissue, preoperative aortic rupture, and usage of preoperative antithrombotic medication. Finally, despite the substantial early and 5-year results of this study, an extended follow-up study should be conducted in the future to analyze the long-term outcomes in patients who underwent DSC procedure.

## Conclusions

Mediastinal packing with DSC is an efficient life-saving technique to stabilize patients who were complicated with intractable bleeding after undergoing ATAAD repair surgery, which leads to acceptable short- and long-term outcomes during a 5-year follow-up. Patients who were at risk for intractable bleeding should be evaluated early to implement DSC.

## Data Availability

The datasets generated and analyzed in the current study cannot be made publicly available for ethical and legal reasons. The Institutional Review Board of Chang Gung Medical Foundation must review any request to share data publicly to protect the patients' privacy. Requests for data can be sent to the Institutional Review Board of Chang Gung Medical Foundation at irb1@cgmh.org.tw.

## Abbreviations

ACP: Antegrade cerebral perfusion
AsAo: Ascending aorta
ATAAD: Acute type A aortic dissection
CI: Confidence interval
CPB: Cardiopulmonary bypass
DSC: Delayed sternal closure
ECMO: Extracorporeal membrane oxygenation
ICU: Intensive care unit
OR: Odds ratio
PSC: Primary sternal closure
RCP: Retrograde cerebral perfusion

## References

[1] Pape LA, Awais M, Woznicki EM (2015). Presentation, diagnosis, and outcomes of acute aortic dissection: 17-year trends from the international registry of acute aortic dissection. J Am Coll Cardiol.

[2] Conzelmann LO, Krüger T, Hoffmann I (2011). German registry for acute aortic dissection type A (GERAADA): initial results. Herz.

[3] Paparella D, Rotunno C, Guida P (2011). Hemostasis alterations in patients with acute aortic dissection. Ann Thorac Surg.

[4] Trimarchi S, Nienaber CA, Rampoldi V (2005). Contemporary results of surgery in acute type A aortic dissection: the international registry of acute aortic dissection experience. J Thorac Cardiovasc Surg.

[5] Liu Y, Han L, Li J (2017). Consumption coagulopathy in acute aortic dissection: principles of management. J Cardiothorac Surg.

[6] Bouboulis N, Rivas LF, Kuo J (1994). Packing the chest: a useful technique for intractable bleeding after open heart operation. Ann Thorac Surg.

[7] Charalambous C, Zipitis CS, Keenan DJ (2002). Outcome of primary chest packing and delayed sternal closure for intractable bleeding following heart surgery. Cardiovasc J S Afr.

[8] Estrera AL, Porat EE, Miller CC (2008). Outcomes of delayed sternal closure after complex aortic surgery. Eur J Cardiothorac Surg.

[9] Hiratzka LF, Bakris GL, Beckman JA (2010). 2010 ACCF/AHA/AATS/ACR/ASA/SCA/SCAI/SIR/STS/SVM guidelines for the diagnosis and management of patients with thoracic aortic disease: a report of the American college of cardiology foundation/American heart association task force on practice guidelines, American association for thoracic surgery, American college of radiology, American stroke association, society of cardiovascular anesthesiologists, society for cardiovascular angiography and interventions, society of interventional radiology, society of thoracic surgeons, and society for vascular medicine. Circulation.

[10] Lin CY, Wu MY, Tseng CN (2020). Surgical rescues for critical hemopericardium complicated by acute type A aortic dissection: emergent subxiphoid pericardiotomy or cardiopulmonary bypass first?. PLoS ONE.

[11] Lin CY, Tseng CN, Lu CH (2020). Surgical results in acute type A aortic dissection with preoperative cardiopulmonary resuscitation: survival and neurological outcome. PLoS ONE.

[12] Lin CY, Tseng CN, Lee HA (2019). Double arterial cannulation strategy for acute type A aortic dissection repair: A 10-year single-institution experience. PLoS ONE.

[13] Lin CY, See LC, Tseng CN (2020). Surgical outcomes analysis in patients with uncomplicated acute type A aortic dissection: a 13-year institutional experience. Sci Rep.

[14] Gielchinsky I, Parsonnet V, Krishnan B (1981). Delayed sternal closure following open-heart operation. Ann Thorac Surg.

[15] Rylski B, Hoffmann I, Beyersdorf F (2014). Acute aortic dissection type A: age-related management and outcomes reported in the German registry for acute aortic dissection type A (GERAADA) of over 2000 patients. Ann Surg.

[16] Sultan I, Bianco V, Patel HJ (2021). Surgery for type A aortic dissection in patients with cerebral malperfusion: results from the international registry of acute aortic dissection. J Thorac Cardiovasc Surg.

[17] Chemtob RA, Fuglsang S, Geirsson A (2020). Stroke in acute type A aortic dissection: the nordic consortium for acute type A aortic dissection (NORCAAD). Eur J Cardiothorac Surg.

[18] Wu HB, Ma WG, Zhao HL (2017). Risk factors for continuous renal replacement therapy after surgical repair of type A aortic dissection. J Thorac Dis.

[19] Helgason D, Helgadottir S, Ahlsson A (2021). Acute kidney injury after acute repair of type A aortic dissection. Ann Thorac Surg.

[20] Mehta RL, Kellum JA, Shah SV (2007). Acute kidney injury network: report of an initiative to improve outcomes in acute kidney injury. Crit Care.

[21] Fanning WJ, Vasko JS, Kilman JW (1987). Delayed sternal closure after cardiac surgery. Ann Thorac Surg.

[22] Boeken U, Assmann A, Mehdiani A (2011). Open chest management after cardiac operations: outcome and timing of delayed sternal closure. Eur J Cardiothorac Surg.

